# Design and performance analysis of a vertically stacked gate-all-around nanosheet FET with embedded nanocavity for biosensing applications

**DOI:** 10.1038/s41598-026-35132-1

**Published:** 2026-01-29

**Authors:** Rudra Lakshmi Prasanna, Srinivasa Rao Karumuri, Vakkalakula Bharath Sreenivasulu, Girija Sravani Kondaveeti

**Affiliations:** 1 VLSI-Microelectronics Research Lab, Department of Electronics and Communication Engineering, Koneru Lakshmaiah Education Foundation (Deemed to Be University, Green Fields, Vaddeswaram, Andhra Pradesh India; 2 Department of Electronics and Communication Engineering, Manipal Institute of Technology Bengaluru, Manipal Academy of Higher Education, Manipal, India

**Keywords:** DM-NSFET, Biosensor, Sensitivity, Vertically stacked, Biophysics, Biotechnology, Materials science, Nanoscience and technology

## Abstract

In this article, we designed and analyzed a Vertically Stacked Gate All Around Dielectric Modulated Nano Sheet Field Effect Transistor (DM-NSFET) based biosensor through TCAD simulations. The DM-NSFET is designed for detection of Cancer biomolecules like SW 620, HEK293, and other biomolecules like DNA, gelatin. This functionality comes out through the modulation of its electrical properties by incorporating cavity all around at two sides of dielectric material (HfO_2_) under the gate electrode to allow biomolecules. The proposed device contains gate all around to increase the sensitivity of device. The sensitivity variation of biosensors is analyzed in terms of subthreshold swing (SS), Selectivity and response time (τ). Further, the effect of filling positions on sensitivity is examined under different cases, this biosensor sensitivity mainly depends on number of biomolecules filling rather than the specific filling position. The obtained results indicates that the proposed device is reaches its current sensitivity of 3.1 × 10^3^, subthreshold swing (SS) of 27.72 mV/dec. The proposed device exhibits significantly enhanced sensitivity compared to existing biosensors and therefore, this biosensor is highly suitable for diagnosis of Cancer Biomolecule.

## Introduction

Now a days, the market for NSFETs is expanding rapidly, it was estimated to be worth USD 214 million in 2024 and is expected to continue growing at a CAGR of about 25% until 2033. The top companies like TSMC, Samsung, and Intel are implementing NSFETs in 3 nm and sub-3 nm nodes because of their approximately 30% increase in performance and power efficiency. According to recent reports, they are essential for enabling edge computing, AI, and IoT applications^1^. The semiconductor industry is focused on CMOS scaling to support high-speed, low-power sensing applications, such as biosensors^2,3^. Biosensors have recently received much attention due to their versatility and impact in various fields such as medical diagnostics, environmental monitoring and agricultural applications^4^. In present days, field effect biomolecules label free recognition method gives more reliable results without changing the characteristics of the biomolecules. FET-based biosensors have drawn a lot of interest among different biosensing techniques because of their scalability, affordability, and capacity to identify charged biomolecules^5,6^. These tools provide a flexible platform for label-free biomolecule detection by translating biological interactions into quantifiable electrical signals^7,8^. However, the traditional FET biosensors to identify neutral biomolecules limit their use. Advanced device architectures, including biosensors based on MOSFETs and Tunnel FETs (TFETs), have been studied to get around this restriction^9–11^.

Although MOSFET-based biosensors have advantages over conventional FETs, such as a higher drain current^12–14^, their practical application is limited by disadvantages, such as higher leakage currents, lower energy efficiency, and an inability to achieve a subthreshold swing (SS) of less than 60 mV/decade^15,16^. However, because of their low power dissipation, increased sensitivity and quicker response times, TFETs, which rely on band-to-band tunneling (BTBT) at the source channel interface have become a compelling substitute for biosensing applications^17,18^. Even with these developments, it is still difficult to detect charged and neutral biomolecules with high sensitivity and selectivity. To overcome this, dielectrically modulated TFET (DMTFET)-based biosensors have been developed, which combine the advantages of TFET technology and dielectric modulation to produce increased sensitivity with fewer short-channel effects (SCEs)^19–27^. These devices also address scaling-related problems that are common in traditional FET-based biosensors, such as hot-electron effects and drain-induced barrier lowering (DIBL). When compared to TFET-based biosensors, the integration of highly scaled three-dimensional devices is thought to be more feasible than depending on traditional planar MOSFETs for FET-based biosensing applications. Researchers have recently shown biosensors based on Gate-All-Around Nanowire FETs and FinFETs^28,29^. Of these, the fully enclosed gate structure used by GAA-NW devices offers a greater gate area in the same footprint than FinFETs, leading to improved gate control capability^30^. GAA-NWFETs can enhance biosensor sensitivity and suppress short-channel effects more effectively thanks to this structure. Significant difficulties are presented by the incredibly small channel width and the intricate fabrication procedure, though^31^. Additionally, the on-state current in GAA-NWFETs is relatively low because of parasitic effects, which restricts their usefulness in biosensing technologies^32^. To improve tunneling efficiency, reduce the ambipolar effect, and attain greater detection sensitivity across a wider range of biomolecules, more developments were required for further advancements^33,34^.

In this paper, for the first time, we have proposed a vertically stacked Gate All Around Dielectric Modulated Nano Sheet FET(DM-NSFET) biosensor to overcome the drawbacks of existing biosensors. The DM-NSFET structure has three stacked channels with the gate length of 10 nm, so it can effectively improve the output characteristics and the nanosheet channel is designed with the length of 35 nm, width of 10 nm and height of 4 nm which improves the ON current and electrostatic control. A cavity all around at two sides of dielectric material (HfO2) is introduced to improve sensitivity and suppress parasitic capacitance. The electrical characteristic of the proposed device is analyzed through drain current, electric field, energy band, I_ON_ and I_ON_/I_OFF_ ratio characteristics. Furthermore, the proposed a Dielectric Modulated Nanosheets FET(DM-NSFET) analyzed the effect of biomolecules, electrical characteristics, cavity engineering, sensitivity analysis for charged and neutral biomolecules^35,36^ and Response Time(τ). The proposed device obtained sensitivity of 3.1 × 10^3^, subthreshold swing of 27.72 mV/dec, these parameters demonstrate that the DM-NSFET is highly suitable for low power-based sensor applications. The proposed DM-NSFET based biosensor analyzed the drain current and sensitivity at different biomolecules like SW 620 at 0.5 THz (Colorectal cancer), HEK293 at 0.5 THz (Kidney cancer), DNA at 900 MHz and Gelatin at 1 MHz for neutral Charge biomolecules. Further, this device analyses the negative charge and positive charge from -1E12 to + 1E12 respectively at V_gs_ = 0.5 V. In this article, device level performance of proposed DM-NSFET have been simulated and analyzed by Atlas TCAD Tools. The main novelty of this work is the development of a vertically stacked DM-NSFET structure that enhances biosensing performance without increasing the device area. In the vertical stacking of nanosheets, the proposed design strengthens the gate-channel electrostatic coupling and increases the effective sensing region inside the nanocavity. The doping-less channel reduces variability and enhances sensing uniformity. This combination enables the device to exhibit a low detection limit, better specificity, and improved linearity compared to earlier NSFET-based biosensors. Overall, the vertical stacking approach introduces a more efficient, highly responsive platform for label-free biomolecule detection.

This paper is organized as follows: The design and dimensions of the proposed device are described in Section II. The findings and explanations of cancer cell detection and analysis are presented in Section III. The work is concluded in Section IV with a summary of the key conclusions and their implications.

## Sensor device structure design parameters, simulation setup and fabrication process

**Fig. 1:**
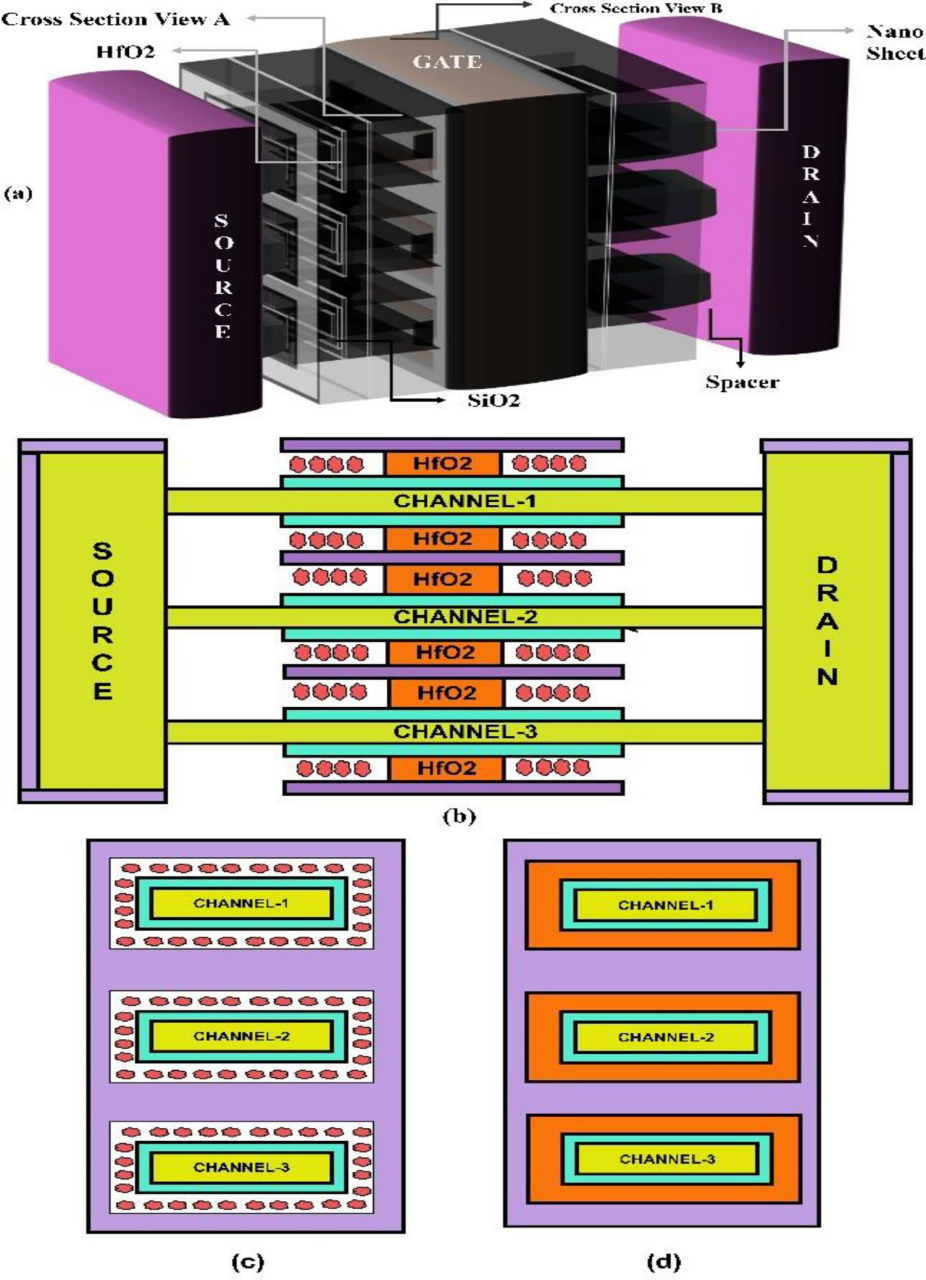
(**a)** 3-dimensional schematic diagram of the proposed DM-NSFET-based biosensor. (**b)** X–Z Direction cross section view. (**c)** Y–Z cross-sectional view B and (**d)** Y–Z cross-sectional view C of DM-NSFET-based biosensor.

**Fig. 2:**
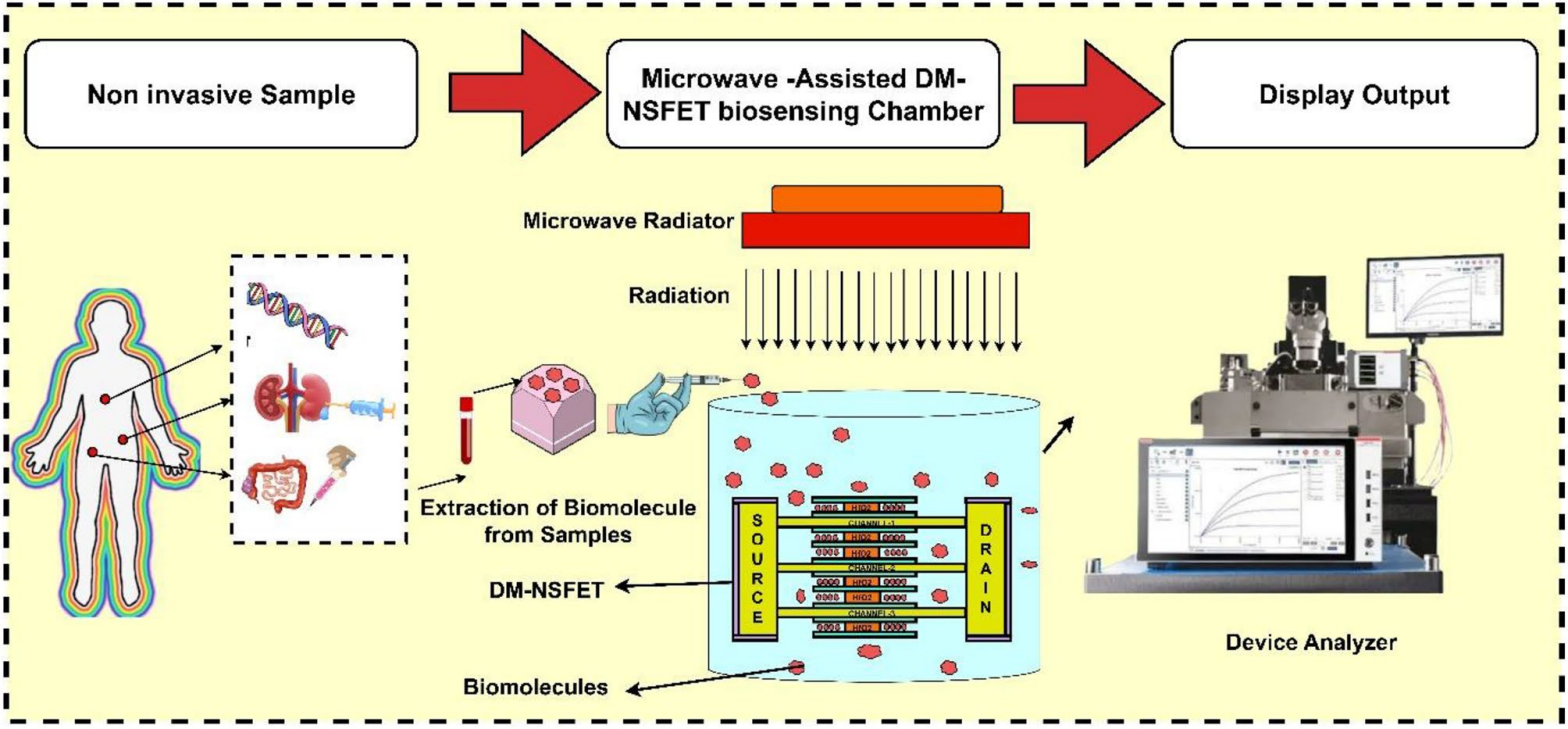
Schematic of biomolecule detection process and measurement setup of proposed Dm-NSFET.

A 3-D schematic of dielectric modulated-NSFET (DM-NSFET)-based biosensor structure is shown in Fig. 1a. The X–Y cross sectional view and the transverse cross-sectional view B and C of proposed biosensor are shown in Fig. 1b,c,d. The dimensions of proposed DM-NSFET parameters are described in Table 1. The nanocavity region between metal gate and nanosheet channel to immobilize target biomolecules. The spacer near the source and drain is removed to immobilize biomolecules within the cavity region easily. The proposed dielectric modulated-NSFET (DM-NSFET) biosensor schematic shows how cancer biomolecules are immobilized is shown in Fig. 2. To reduce leakage currents and suppress short-channel effects, the device uses silicon material across the source, channel, and drain regions. To detect bio-analytes, two nanocavities are incorporated under gate metal. The positioning of nanocavity improves the biosensor’s sensitivity and selectivity for cancer biomolecules. Biomolecules bind selectively to the Au-linker-functionalized surface, ensuring equilibrium-based specific adsorption rather than only electrostatic isoelectric effects. Hence, the device operates as a selective biosensor, not an ion-sensitive FET. The proposed DM NSFET design has features that minimize noise and molecular interference. Strong electrostatic control through the surrounding-gate structure reduces interface-trap fluctuations, hence suppressing low-frequency noise. The selective receptor layer coating on the sensing cavity limits nonspecific binding and the resultant disturbances from unrelated biomolecules. It also provides stability to the local charge environment so that the device can retain a clear and distinguishable response even in the presence of molecules that may come in with different permittivity or charge states. These combined effects enhance stability and interference tolerance of the sensor^37,38^.

**Table 1:** Simulation parameters and device parameters of DM-NSFET.

Parameter	Values
Gate length (L_g_) (nm)	15
Drain length (L_d_) (nm)	10
Spacer length (nm)	5
Nanosheet thickness(nm)	4
Nano sheet width (nm)	10
Length of cavity (L_cavity_) (nm)	10
Work function of gate, Φ_g_	4.9
Work function of source, Φ_s_	4.1
Work function of drain, Φ_d_	3.1
Thickness of cavity (T_cavity_) (nm)	4
Spacing between NS (nm)	10
Cavity fill (%)	25, 50, 75, 100
Dielectric constant of biomolecule (K)	3.9, 4, 6.1, 12
Charge densities of biomolecule	− 1E12 TO + 1E12
Si thickness (nm)	10
BTBT (non-local) tunneling model	Enabled
TAT with trap density (N_T)	1 × 10^19^ cm⁻^3^
Bandgap narrowing (BGN)	Enabled
SRH recombination (τₙ, τₚ)	1 × 10^–6^ s
SiO₂ dielectric constant (under M1/M2)	3.9
HfO₂ dielectric constant (under M3)	25

The selective binding with target molecules is facilitated by the localized nature of the nanocavities, and this interaction results in detectable changes in the electrical characteristics of the device. In liquid-phase sensing, ions at the oxide–electrolyte interface form an electric double layer (EDL) that screens the biomolecule charge according to the Debye length (< 1 nm for physiological buffers). Introduce a commonly used semi-realistic approach without performing a full Poisson–Boltzmann EDL simulation, which would be computationally prohibitive for 3D TCAD. The screening effect in an effective dielectric constant (K) representing the biomolecule–functionalization–buffer environment. A thin functionalization layer is assumed (APTES + cross-linker, ~ 1–2 nm), which positions the biomolecule within the Debye length. This simplified model maintains numerical stability and computational efficiency while still capturing the essential electrostatic behavior. As a result, the proposed biosensor makes it possible to identify and measure cancer biomolecules.

**Fig. 3:**
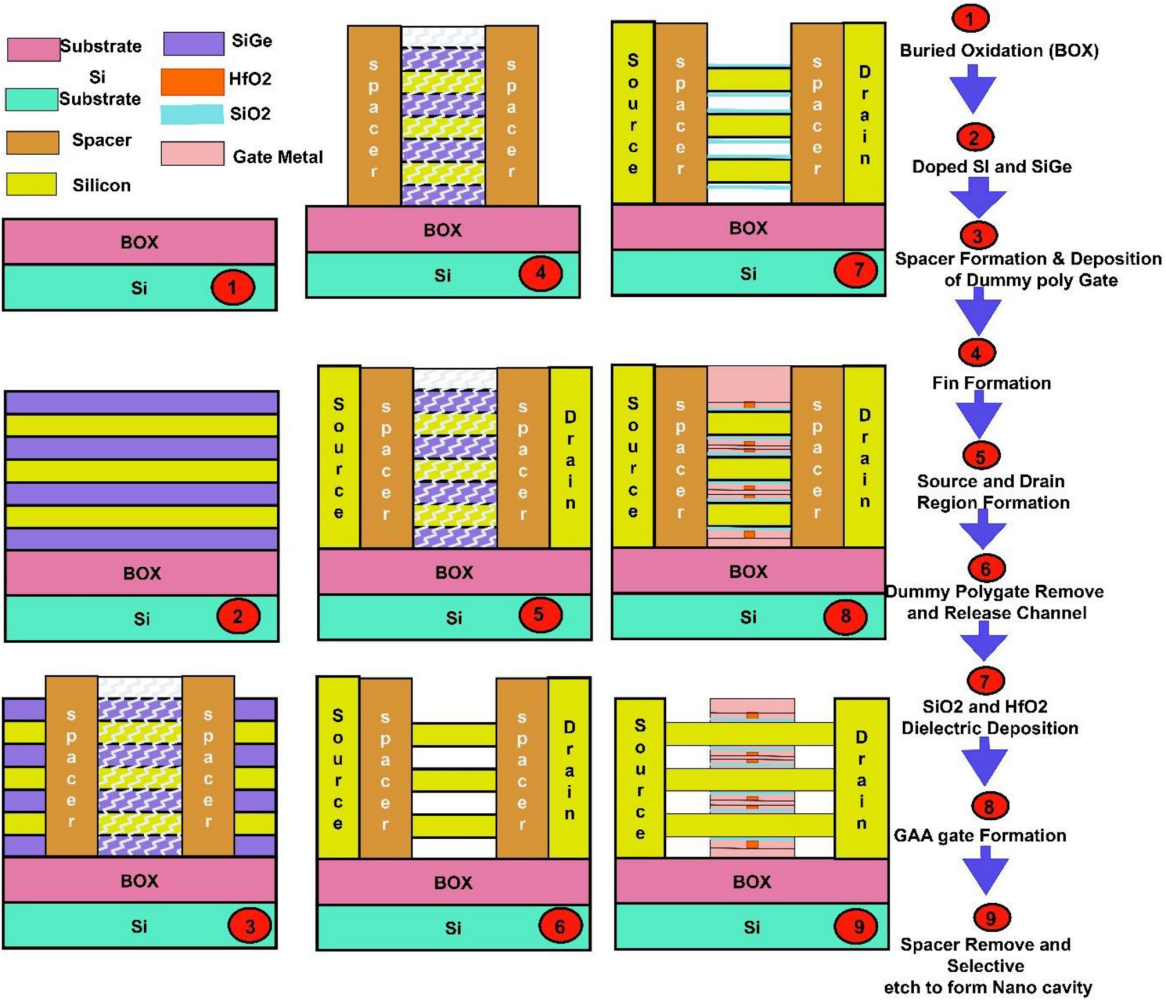
Tentative fabrication flow.

The fabrication process of the proposed DM-NSFET is shown in Fig. 3. The process begins with a commercial SOI wafer. The buried oxide (BOX) layer is formed by thermal oxidation at 1000–1050 °C, which is a standard step in DM-NSFET manufacturing. This stage involves no special complexity. A thin Si and SiGe stack is deposited using conventional LPCVD (550–650 °C) or MBE (~ 500 °C). These epitaxial processes are widely used in modern CMOS technologies for strain-engineering and therefore do not introduce additional fabrication challenges. Doping is achieved using ion implantation followed by RTA at 950–1000 °C for 10–30 s. Both techniques are well-established and compatible with standard fabrication lines. Since the proposed device is dielectric-modulated, no ultra-high doping precision is required, reducing complexity. SiO₂/SiN spacers are deposited by PECVD at 350–400 °C and patterned using anisotropic RIE. These are routine steps in Dm-NSFET processes. A polysilicon dummy gate is deposited by LPCVD (~ 620 °C) and patterned through DUV lithography. Dummy-gate processes are commonly used in HKMG (high-k metal gate) technologies, contributing no unusual difficulty. The fin is patterned using DUV lithography and anisotropic dry etching (chamber temperature ~ 200 °C). This step is standard across 14 nm/10 nm NSFET nodes and does not add novelty-specific complexity. Source and drain regions are formed through selective epitaxy at 600–700 °C or high-dose implantation followed by RTA (1000–1050 °C). Both routes align with conventional CMOS flows. The dummy gate is removed using TMAH wet etching (~ 85 °C), which exposes the channel region. This step is identical to the replacement-gate process in HKMG fabrication. A thin SiO₂ interface layer is thermally grown at 800–850 °C, and HfO₂ is deposited by ALD at 250–300 °C. A TiN/W metal gate is deposited using ALD/PVD (300–400 °C). These steps are standard in DM-NSFET technology. After gate definition, a controlled etching sequence removes selective spacer regions through anisotropic dry etching (~ 200 °C), followed by mild wet etching at 80–90 °C to create nanocavities under the source region. The cavity dimensions are comparable to underlap recesses used in advanced DM-NSFET fabrication. The technique relies on selective etch-stop layers, preventing damage to the channel. This is the only fabrication step requiring additional precision, but similar processes have already been demonstrated for dielectric-modulated biosensor structures. The etched cavities serve as bio-recognition where analytes with different dielectric constants modulate the tunneling characteristics. No post-CMOS biological processing is required beyond surface functionalization.

The ATLAS Silvaco TCAD is used to analyze the device’s performance, capturing nanoscale transport mechanisms with advanced models. Band-to-band tunneling at the source–channel interface was accurately simulated using the non-local BTBT model.

**Fig. 4:**
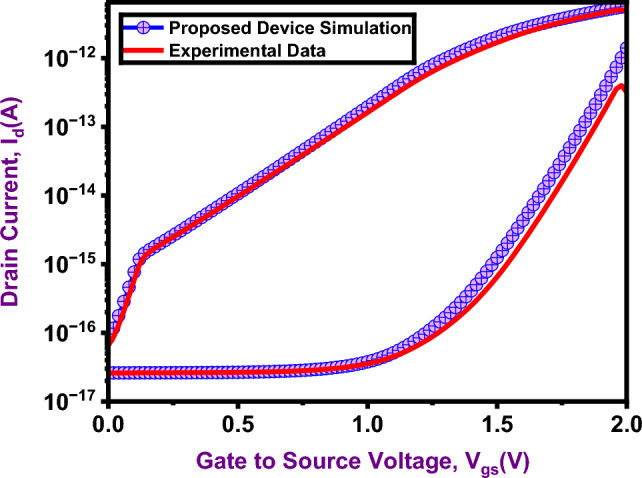
Calibration of TCAD model with simulated data^39^.

TAT was enabled explicitly by using the TAT tunneling model in ATLAS, and SRH recombination was included to model thermal trap-assisted generation and recombination. The degradation of carrier mobility was modeled by using the CVT approach, while BGN was incorporated to account for heavy doping effects. Additionally, a field-enhanced SRH model was used, accounting for trap interactions that depend on electric fields. Strong agreement was found between the simulated and experimental results, as evidenced by the drain current–gate voltage characteristics (Fig. 4). Table 1 provides specifics on the electrical parameters and optimized device geometry.

## Results and discussions

This section presents the simulation results of the DM-NSFET biosensor under exposure to biomolecules with dielectric constants of K = 3.9,4, 6.1 and 12. The analysis investigates the variations in I_ON_/I_OFF_ ratio, selectivity and overall sensitivity for both neutral and charged biomolecules. Furthermore, the study assesses device behavior when the nanogap cavities are partially filled and examines the effect of steric hindrance, demonstrating that the sensor maintains reliable detection across diverse operating conditions.

### Impact of cavity geometry

**Fig. 5:**
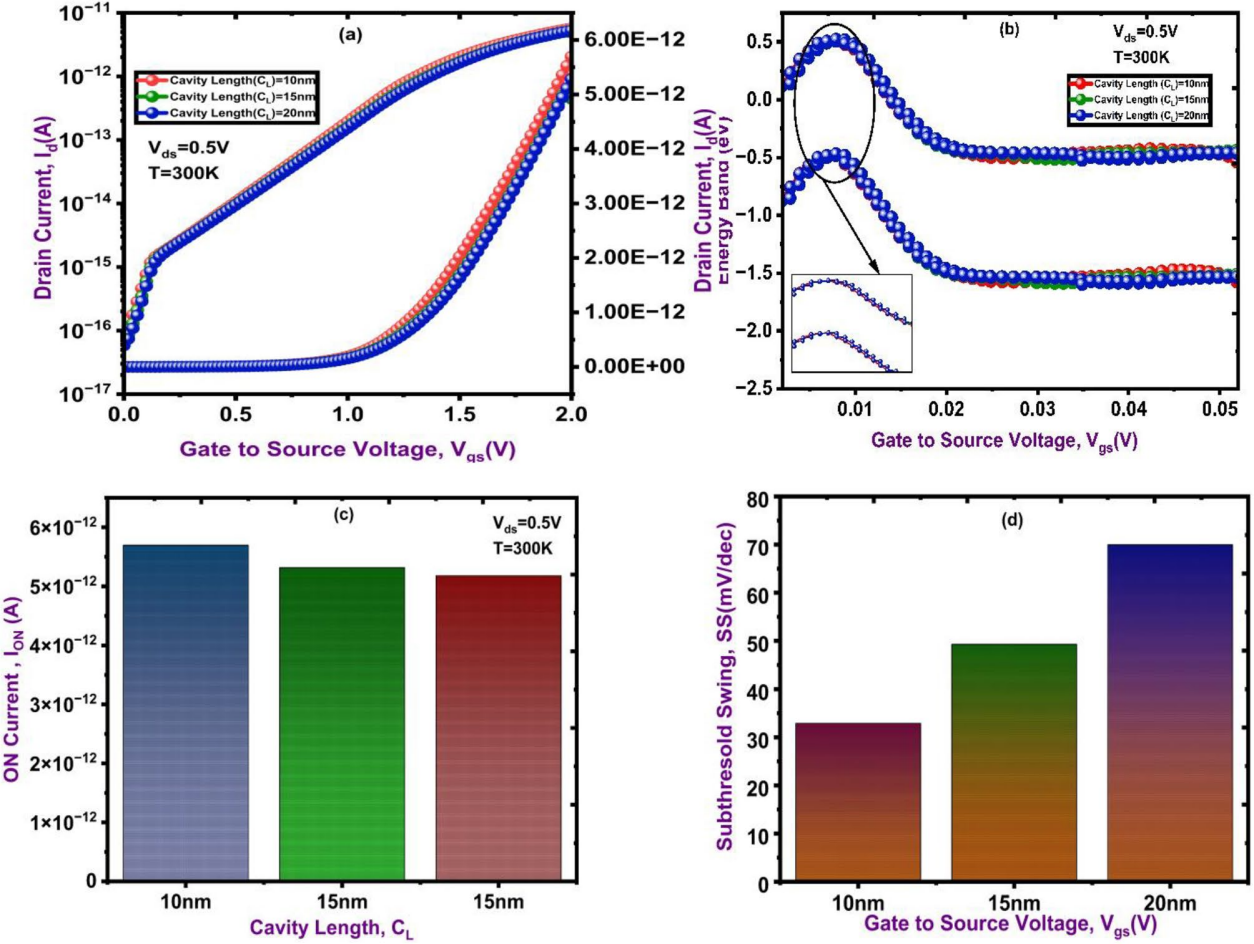
Transfer characteristics of DC DM NSFET at different cavity lengths. (**a)** Drain Current, (**b)** Energy Band, (**c)** I_ON_ Current, (**d)** Subthreshold Swing.

In the DM-NSFET biosensor, cavity length engineering is a factor in determining performance by affecting the extent gate modulation. Stronger gate control and improved capacitive coupling develops carrier accumulation at the smallest cavity length of 10 nm, where the drain current is highest, as shown in Fig. 5a. On the other hand, greater cavity lengths obtained less current flow because they lessen the impact of the electric field. This demonstrates that the device’s sensitivity and current conduction are greatly enhanced by reducing the cavity length. Figure 5b shows the DM-NSFET’s energy band diagrams for various cavity lengths. The conduction band bends more efficiently for the 4 nm cavity length, making it simpler to inject carriers into the channel. Conversely, larger cavities result in less effective modulation because they limit the gate’s ability to control the channel potential. Consequently, stronger electric field coupling and improved band bending are made possible by the 10 nm cavity length structure, which improves carrier transport. The I_ON_ at different cavity lengths is displayed in Fig. 5c. The High I_ON_ is obtained at 10 nm cavity length, because of the enhanced electrostatic interaction between the gate and the channel. Current flow is reduced by fringe effects and leakage paths at by large cavity lengths. This demonstrates that increasing sensing signals requires decreasing cavity length. Lastly, the subthreshold swing (SS) for various cavity lengths is shown in Fig. 5d. The 10 nm cavity length has the lowest SS, which suggests stronger gate control and sharper switching behavior. Higher SS values and slower device response are observed at large cavity lengths. Improved electric field penetration and faster switching are confirmed by the reduced SS in the 10 nm cavity, increasing the device’s sensitivity and responsiveness for biomolecule detection.

**Fig. 6:**
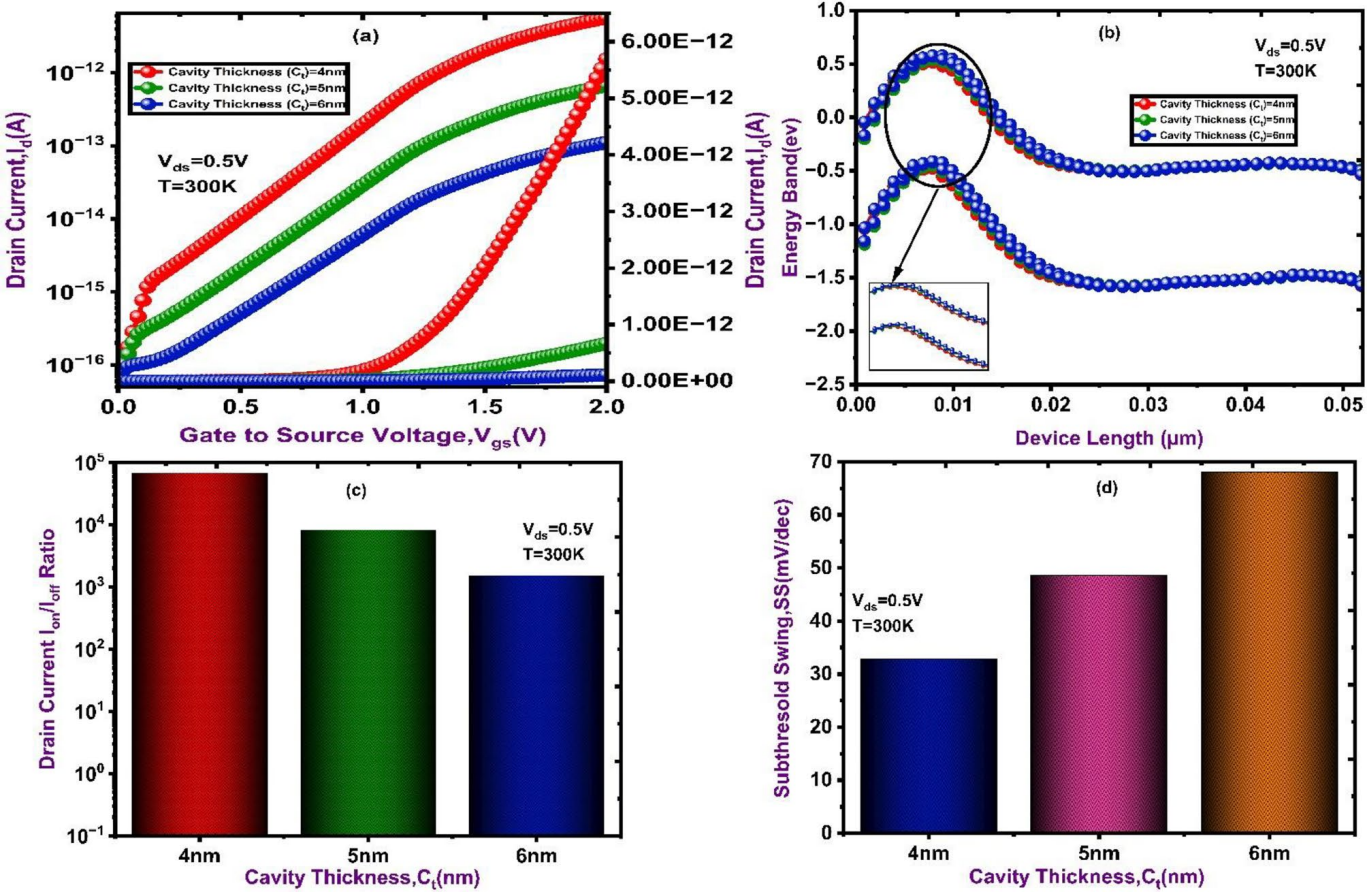
Transfer characteristics of DM NSFET at different cavity thickness. (**a**) Drain Current (**b**) Energy Band (**c**) I_ON_/I_OFF_ Ratio (**d**) Subthreshold Swing.

Cavity thickness plays a vital role in determining the strength of gate control and the efficiency of band-to-band tunneling (BTBT). In Fig. 6a, the drain current increases significantly across the gate voltage range when the cavity thickness is reduced from 6 to 4 nm. This improvement is explained by the ferroelectric gate’s ability to induce a higher electric field at the tunneling junction due to the stronger electrostatic coupling between the gate and the channel as the dielectric gap narrows. Better carrier tunneling is made possible by the energy band diagrams shown in Fig. 6b, which displays a more abrupt band transition in shorter cavities. The drain current Ratio (I_ON_/I_OFF_) is highest for the smallest cavity thickness of 4 nm, indicating better switching efficiency and stronger gate modulation. As the cavity thickness increases from 4 to 6 nm, the ratio decreases due to weaker electric field control and reduced sensitivity is shown in Fig. 6c. The subthreshold swing (SS) is lowest for the smallest cavity thickness of 4 nm, indicating sharper switching and improved gate control is shown in Fig. 6d. As the cavity thickness increases from 4 to 6 nm, the SS value rises, reflecting degraded switching performance and reduced sensitivity.

### Electrical characteristics of proposed DM-NSFET

**Fig. 7:**
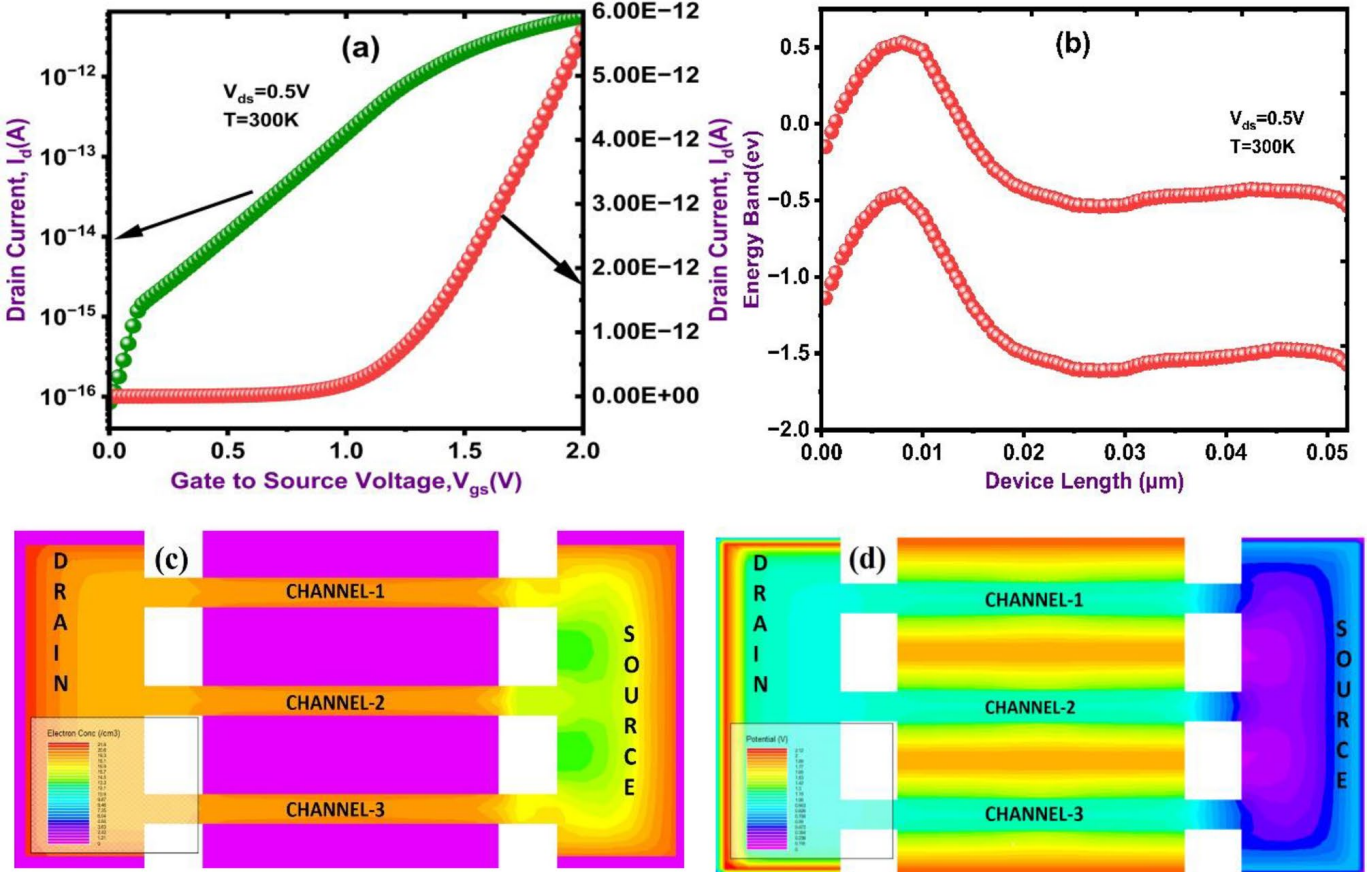
Transfer characteristics of DC DM NSFET (**a**) drain current (**b**) Energy Band (**c**) Electric Concentration (**d**) Potential contour of DM NSFET.

The electrical performance of proposed Nanosheet FET with two-sided cavities surrounded by each channel are shown in Fig. 6. Strong gate modulation made possible by the cavity structure (length is 10 nm and Thickness is 4 nm) causes the drain current (I_d_) to rapidly increase at a particular threshold voltage is shown in Fig. 7a, which displays sharp switching behavior. The energy band variation along the device is shown in Fig. 7b, where efficient band-to-band tunneling (BTBT) fueled by the stronger electric field in smaller cavities is highlighted by reduced bandgap regions close to the source-channel interface. The electron concentration in Fig. 7c shows that carriers are efficiently injected into all three channels, with peak concentrations near the source close to gates. The device’s potential distribution is uniformly distributed which is shown in Fig. 7d, which lowers leakage and develops steady current conduction. By enabling the electric field to envelop the channel and reduce short-channel effects, the surrounding cavities improve gate control. The adsorption of biomolecules follows concentration-dependent binding kinetics, reaching equilibrium as the surface saturates. This charge modulation alters channel potential and current, confirming a reaction-limited transport mechanism. Additionally, by increasing field intensity at the tunneling junction, this design minimizes subthreshold leakage and increases carrier transport. Higher ON current, enhanced switching performance, and more effective energy band modulation are the results of these advancements.

### Effect of biomolecules

**Fig. 8:**
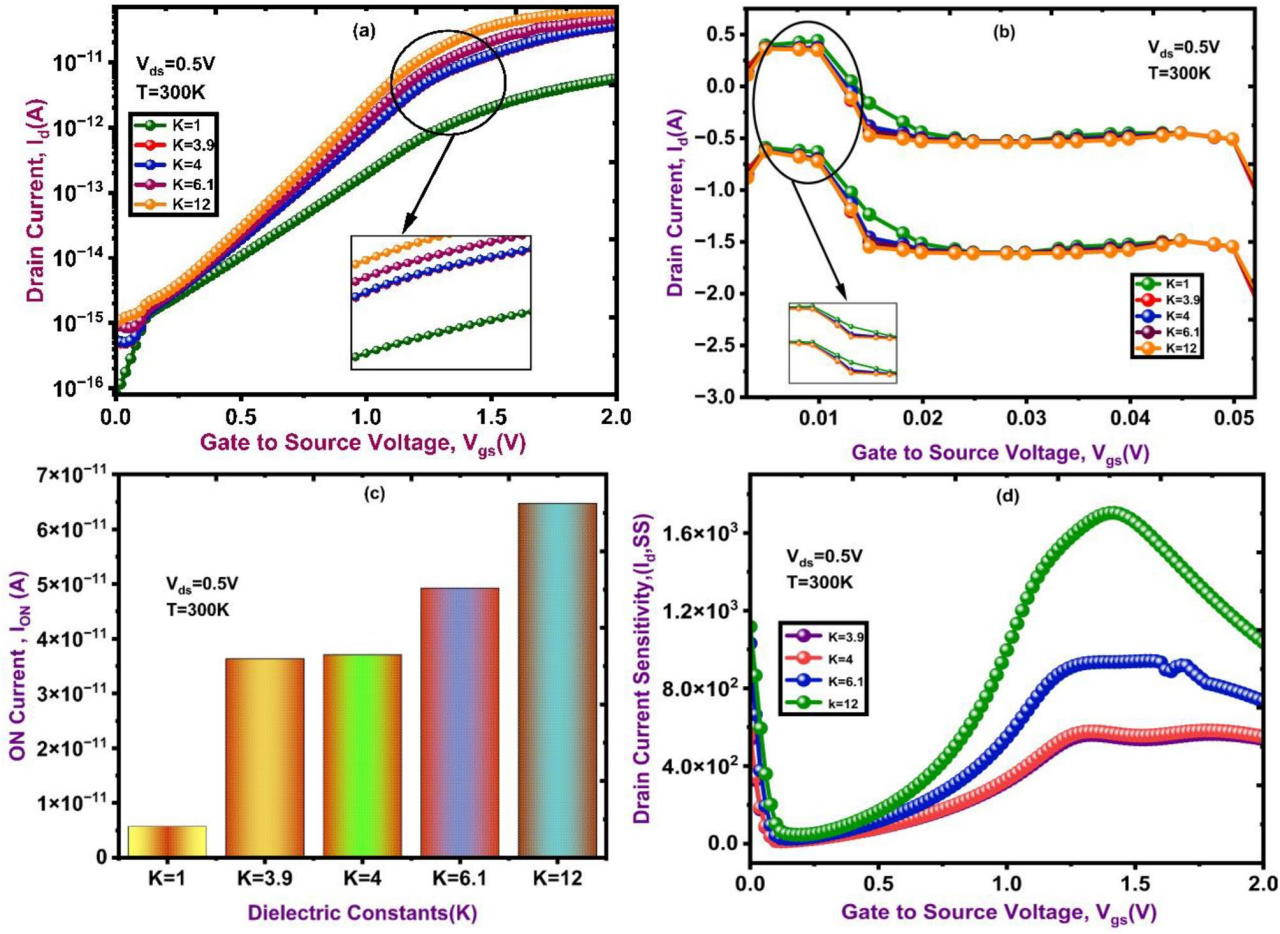
Transfer characteristics of DM-NSFET for different biomolecules. (**a**) Drain Current (**b**) Energy Band (**c**) Ion Current (**d**) Drain Current Sensitivity.

This section presents the simulation outcomes of the proposed DM-NSFET biosensor, focusing on cancerous cell detection as summarized in Table 2. The study examines how the immobilization of various cancer biomarkers influences the drain current characteristics and modulates the energy band profile. Biomolecules with neutral charge for detecting non-ionic species at different K-values are shown in Fig. 8.The drain current exhibits a steeper rise in relation to the gate-to-source voltage (V_gs_) and increases significantly with higher dielectric constants (K-values) is shown in Fig. 8a. This behavior points to a stronger gate-channel coupling, which would improve sensor sensitivity by enhancing charge induction even in areas with neutral biomolecules. Additionally, the energy band bending increases as the K-values rise, this indicates better modulation of the channel’s energy states.

**Table 2:** Simulation dielectric frequency and operating frequencies of cancer cell lines.

Biomolecules	Dielectric constant	Frequency
SW 620	3.9	0.5 THz
HEK 293	4	0.5 THz
DNA	6.1	900 MHz
Gelatin	12	1 MHz

. As shown in Figure. (b), this leads to more efficient charge carrier separation and transport, which improves the biosensors’ performance. According to these results, it is crucial to optimize dielectric characteristics to increase sensitivity and detection efficiency, especially when determining the concentrations of neutral biomolecules in biomedical applications. The Fig. 8c shows that the ON current (I_ON_) ​increases with an increase in the dielectric constant (K), indicating improved gate control and reduced leakage across a range of dielectric Constants (K = 3.9 to 12). High-K dielectric constant values significantly enhance device performance when compared to conventional low-K dielectric constant values. Figure 8d shows that as the dielectric constant (K) rises, the sensitivity to drain current increases, reaching its maximum response at K = 12. The high-K dielectrics improve the device’s sensitivity and overall functionality.

### Sensitivity comparison

The drain current sensitivity of the DM-NSFET biosensor has been analyzed and compared with that of a TFET, a conventional GAA-NSFET (Conv. NSFET), and the proposed DM-NSFET.

**Fig. 9:**
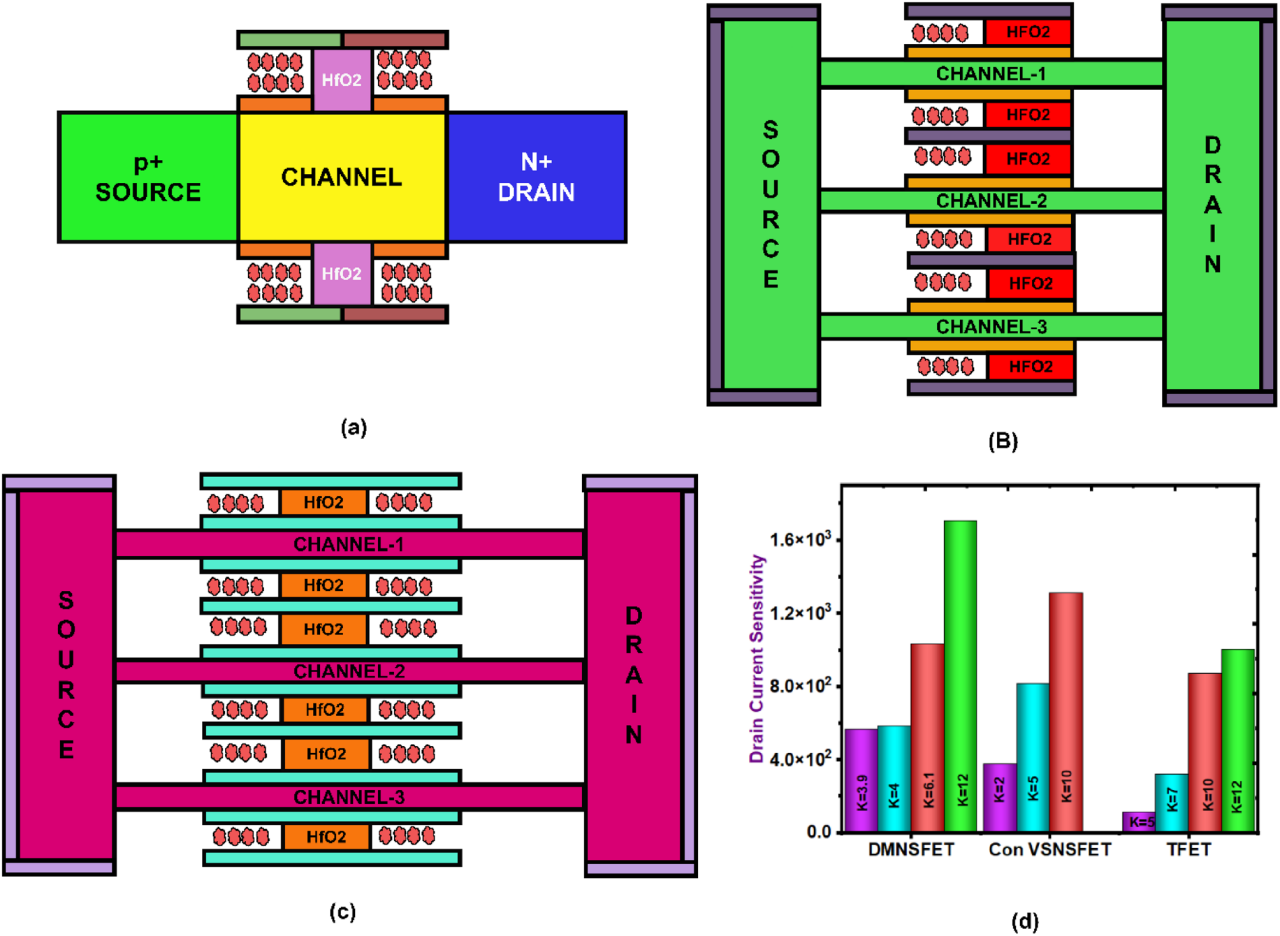
Two-dimensional cross section view of (**a**) TFET, (**b**) Conventional GAA NSFET biosensor, (**c**) DM-NSFET biosensor, and (**d**) comparison of current sensitivity.

Figure 9a–c shows the schematic architectures of these devices. Figure 9d shows that the proposed DM-NSFET achieves superior drain current sensitivity compared to the other structures. This enhancement is attributed to its innovative design, where the sensing cavity is strategically placed near the source and drain regions. The proposed device uses dual nanocavities, this configuration provides stronger dielectric modulation and better channel coupling than a single cavity. A dual-cavity arrangement is more compatible with vertically stacked structure leads to improve sensitivity and uniform biosensing performance.

### Sensitivity analysis

Biomolecules with neutral or charged are essential for identifying non-ionic species that are mainly diagnostic and research Oriented, such as hormones, medications and metabolites. Then detection improves sensitivity and permits in-depth examination of structural dynamics and biomolecular interactions in biological systems. This capability extends the application of biosensor can used in fields like pharmaceuticals, food safety and environmental monitoring. Among biosensing Technologies, nanoscale semiconductor-based biosensors are the most widely used biosensing technology in the biomedical industry due to exceptional sensitivity. The ability of a biosensor to precisely detect and measure substance concentrations is reflected in its sensitivity, which is defined as its capacity to recognize and react to biological targets or analytes. High sensitivity must be attained to guarantee accurate and dependable measurements. The proposed DM-NSFET biosensor is specifically designed and optimized to maximize sensitivity, which can be calculated using Eq. (1):

1 $$Sensitivity = \frac{{I_{d} \left( {bio} \right) - I_{d} \left( {air} \right)}}{{I_{d} \left( {air} \right)}} \times 100$$

**Fig. 10:**
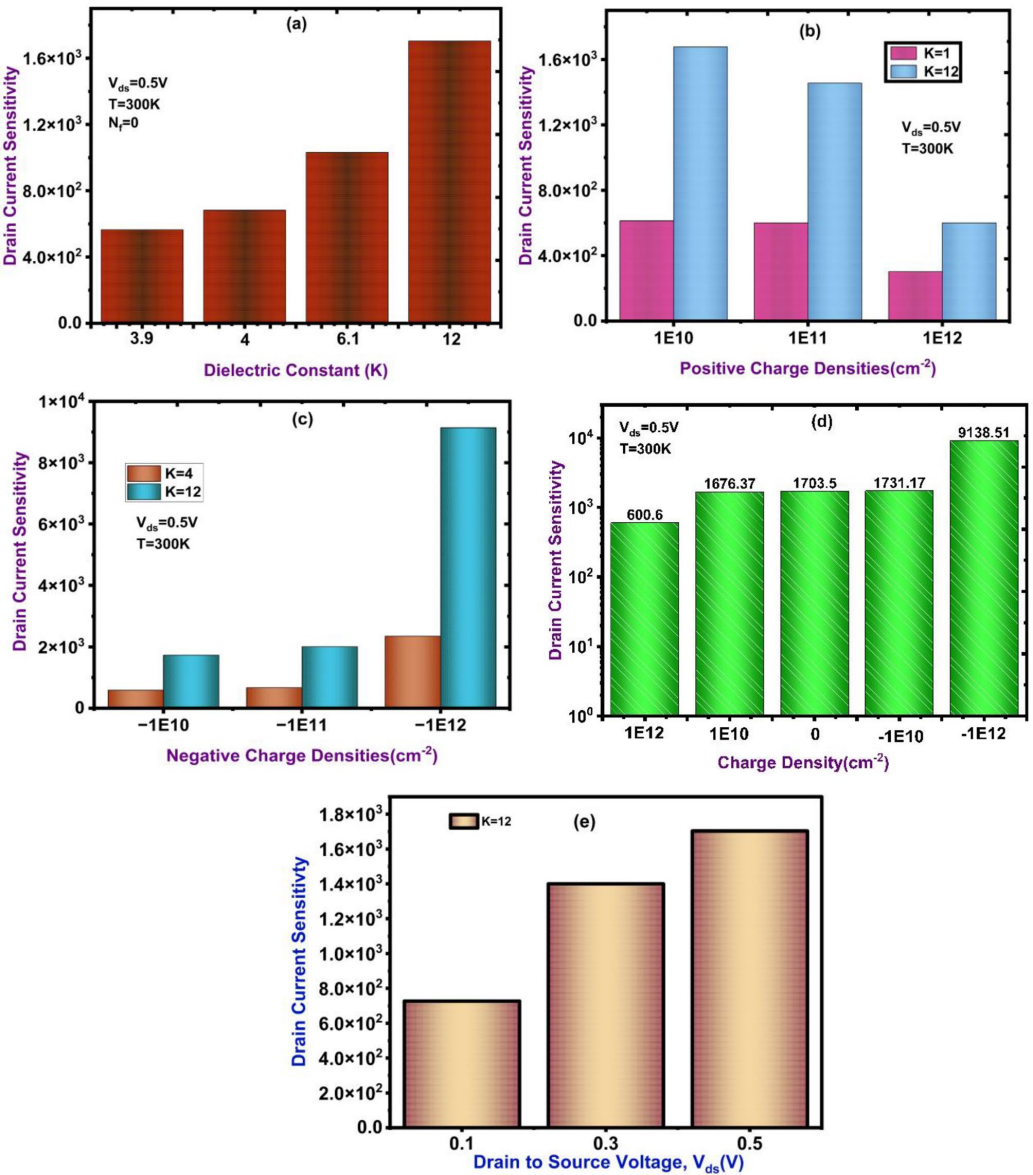
(**a**) Neutral charge density drain current sensitivity (**b**) Positive charge Density drain current sensitivity (**c**) Negative charge Density drain current sensitivity (**d**) Neutral and Charge Density drain current sensitivity (**e**) Drain current sensitivity at different V_ds_ Voltages.

The proposed DM-NSFET biosensor’s sensitivity evaluation is shown in Fig. 10. The response for neutral biomolecules with varying dielectric constants is shown in Fig. 10a, where the sensitivity increases with dielectric constant and high at K = 12. This shows how stronger gate-to-channel coupling and improved charge induction are two ways that higher dielectric biomolecules improve sensor response. The sensitivity behavior for positively charged biomolecules at surface charge densities ranging from 1E10 to 1E12 C/cm^2^ for K = 4 and K = 12 is shown in Fig. 10b. Stronger gate control at higher dielectric constants enhances the biosensor’s capacity to identify positive charge variations, as evidenced by the results, which show consistently higher sensitivity. The response for negatively charged biomolecules charge density -1E12 to -1E10 and dielectric conditions is similarly shown in Fig. 10c. In this instance, the sensitivity is marginally higher, indicating that, in contrast to positive charges, negative charges enable stronger surface potential modulation and more efficient tunnelling. The Fig. 10d shows how drain current sensitivity varies with charge density, where negatively charged biomolecules exhibit greater sensitivity than neutral or positively charged ones.

This suggests that negative charges strengthen tunneling and gate-to-channel coupling, leading to enhanced sensor response. The drain current sensitivity changes with respective drain to source voltage (V_ds_), higher sensitivity obtained at V_ds_ = 0.5 V. Sensitivity increases from values of 7.27 × 10^2^ at V_ds_ = 0.1 V, to 1.40 × 10^3^ at V_ds_ = 0.3 V, and reach a maximum of 1.70 × 10^3^at V_ds_ = 0.5 V. This very significant enhancement confirms that higher drain bias increases carrier acceleration, reduces the effective tunneling barrier thickness and amplifies current variation due to small environmental changes, which is much needed in a high-performance sensing application.

**Fig. 11:**
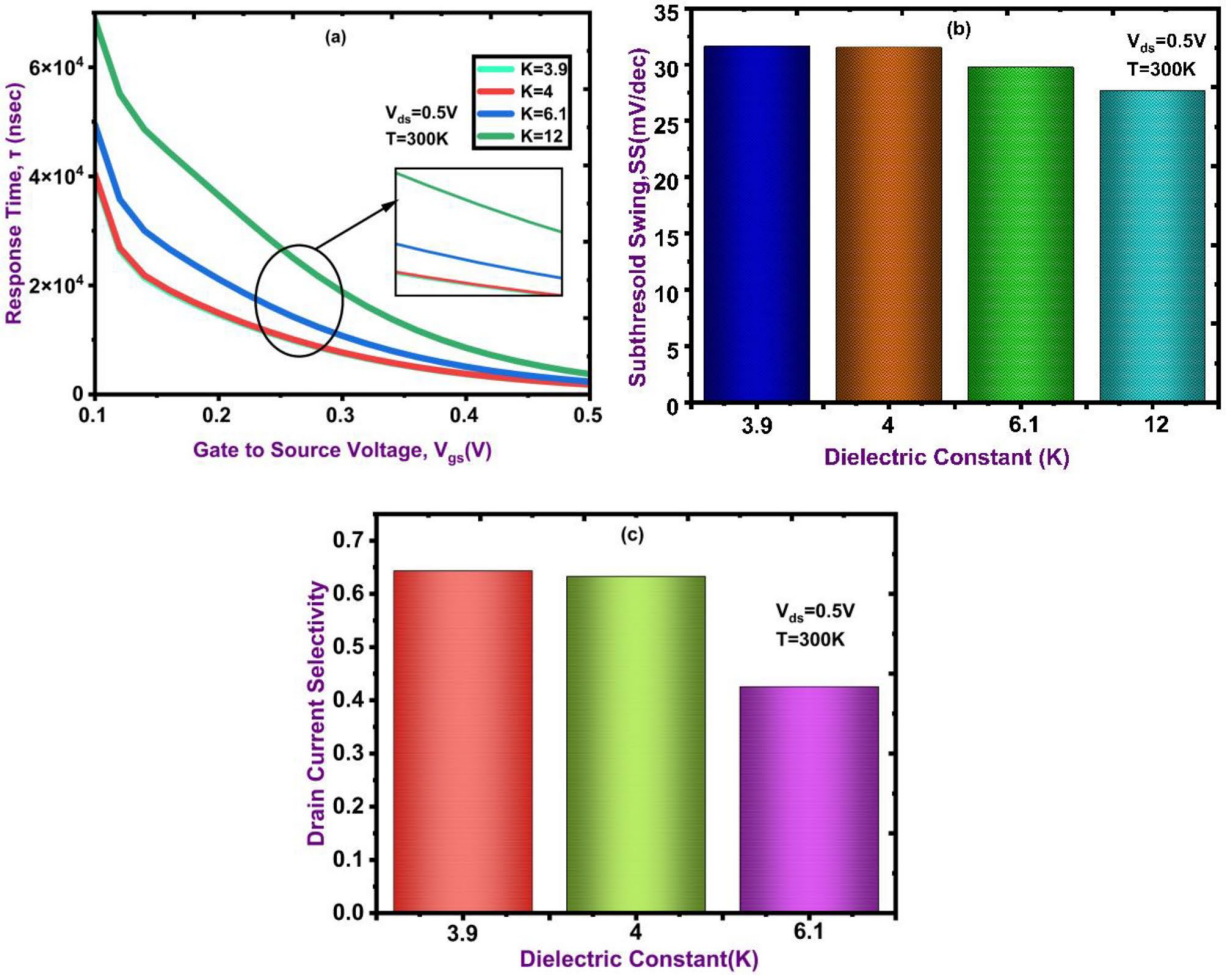
(**a**) Response Time as a function of gate-to-source voltage (V_gs_), (**b**) variation of subthreshold swing (SS) with dielectric constant (*K*), and (**c**) drain current selectivity for different dielectric constants.

The response time is an essential feature for biosensors, especially for applications rapid and real-time detection. The following expression is used to calculate response Time:

2 $$\tau = \varepsilon \sigma$$

where σ indicates the surface conductance and ε is the permittivity of the dielectric material (including both HfO₂ and the dielectric of the target biomolecule). Figure 11b shows how response time varies with gate voltage (V_gs_) for dielectric constants (K = 3.9, 4, 6.1, and 12), which represents distinct biomolecular targets. As V_gs_ increases, the response time decreases, indicating lower RC time constants at higher gate voltages and faster carrier modulation. Furthermore, biomolecules with larger dielectric constants (e.g., K = 12) show noticeably faster response times because of stronger polarization effects and improved surface interactions. Figure 11c shows how the dielectric constants (K = 3.9,4,6.1, and 12) affect the subthreshold swing (SS). The tunneling drain current in the proposed DM NSFET is analytically modeled using the Kane/WKB approximation, where the BTBT generation rate is expressed as

3 $$G\left( E \right) = AE^{2} exp\left( { - \frac{B}{E}} \right)$$

where $B = \frac{{\pi \sqrt {2m^{*} } E_{g}^{3/2} }}{3qh}$,A is a material constant, E is the local electric field at tunnelling junction, m* the effective mass and E_g_ the band gap. For a uniform field the drain current expressed as Eq. (4)

4 $$I_{D} \propto E^{2} exp\left( { - \frac{B}{E}} \right)$$

Indicating that the tunnelling current strongly depends on electric field magnitude. In proposed device structure, the local electric field E at tunnelling junction is controlled by the effective capacitance C_ox,eff,_ which dependence on the nanocavity dielectric є_,bio,_ explain the strong dielectric sensitivity observed in TCAD. The TFET subthreshold swing follows

5 $$SS = \frac{{ln\left( {10} \right)}}{{\left( {\frac{2}{E} + \frac{B}{{E^{2} }}} \right)\frac{dE}{{dV_{g} }}}}$$

Thus, a large $\frac{dE}{{dV_{g} }}$ or larger E reduces SS. Dielctric modulation that increases C_ox,eff,_ increases $\frac{dE}{{dV_{g} }}$, thereby reducing SS, consistent with your simulation showing the proposed device has lower SS in the proposed DM-NSFET. As K increases, SS decreases, suggesting improved gate control and reduced leakage. These findings show that in the subthreshold region, high-K dielectrics greatly increase device efficiency. The following expression is used to calculate Subthreshold Swing (SS):

6 $$SS = \frac{{dV_{GS} }}{{d\left( {\log_{10} I_{D} } \right)}}$$

where, V_gs_ = Gate to Source Voltage, I_D_ = Drain Current. The drain current selectivity at various dielectric constants (K = 3.9,4, and 6.1)^39^. Selectivity is found to be slightly lower at K = 6.1 and higher for K = 3.9 and K = 4. The selectivity of drain current is calculated by following Eq. (7) ^22^:

7 $$Selectivity = \frac{{I_{d} \left( {K = 12} \right) - I_{d} \left( {K = 3.9, 4, 61} \right)}}{{I_{d} \left( {K = 12} \right)}}$$

The proposed device selectivity is highly influenced by dielectric constant, with lower-K values providing superior discrimination ability.

### Benchmarking effect of cavity filling factor and steric hindrances issues

The occupancy factor has a significant impact on the tunneling process in sensing applications. There is less tunneling when the occupancy factor is low because there are fewer states filled in the conduction band. In this analysis, tunneling must be started with a higher threshold voltage and a stronger electric field. Higher occupancy factors facilitate lower the threshold voltage by developing electron tunneling across the barrier. The following formula is used to calculate occupancy of factors^22^:

8 $$\gamma_{bio} = \frac{Thickness\; of\; Cavity\; filled}{{Total\; Thickness \;of \;Cavity }} \times 100$$

**Fig. 12:**
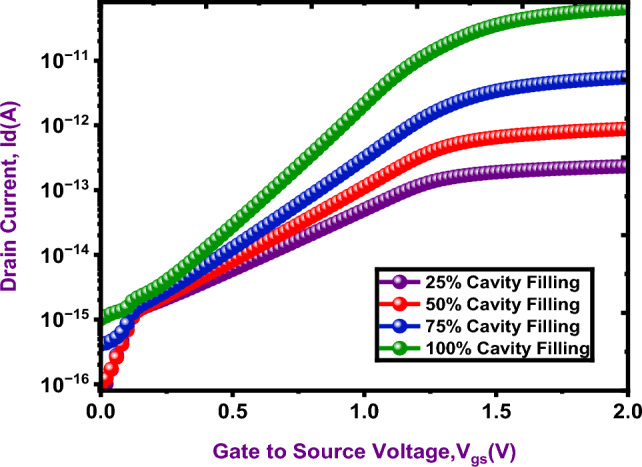
Cavity occupancy-drain current at K = 4.5

A higher drain current results from increasing the occupancy factor, which also improves tunneling efficiency is shown in Fig. 12.

More effective charge transfer across the tunnel junction is made possible by the increase in charge carriers within the conduction band. As a result, the occupancy factor greatly increases sensitivity and overall performance, making the proposed DM-NSFET biosensor more suitable for applications. The sensitivity of the DM-NSFET biosensor was examined by considering nanocavity was completely (100%) filled with biomolecules. Nevertheless, the sensitivity decreases when the cavity is partially filled. Therefore, it is crucial to assess sensitivity in relation to fill factor (FF) to gain a better understanding of the device’s performance limitations. Furthermore, sensitivity is also influenced by the spatial arrangement of biomolecules within the nanocavity. The response of the biosensor under various FF values and random biomolecule distributions within the nanocavity were examined to investigate Steric Hindrances Issues. The sensitivity results of the proposed NS-TFET biosensor are shown in Table 3 for various FF levels and biomolecule configurations, considering both neutral and charged (positive and negative) biomolecules at a dielectric constant of K = 12. For this assessment, each nanocavity was divided into ten regions, each 1 nm wide. In Table 3, the white areas indicate empty spaces filled with air (K = 1), while the sky blue areas indicate regions filled with biomolecules (K > 1). The findings show that higher sensitivity is produced by biomolecules positioned closer to the tunneling junction. For example, even though the FF in cases 5 and 6 is 60%, case 6 sensitivity is higher due to the biomolecules closer proximity to the tunneling junction.

**Table 3:** Sensitivity of the proposed biosensor for different cases of biomolecule placement within a nanocavity.

No. of cases	Sky blue shaded area represents the habitation of biomolecules cavity’s occupied area (%)	Positive charge biomolecules (K = 12, Nf = 1E12)	Neutral biomolecules (K = 12, Nf = 0)	Negative charge biomolecules (K = 12, Nf = -1E12)
		S_id_	S_id_	S_id_
1	20	1.58 × 10^01^	3.18 × 10^02^	9.75 × 10^02^
2	20	2.13 × 10^01^	3.94 × 10^02^	1.04 × 10^03^
3	40	2.17 × 10^01^	4.43 × 10^02^	2.30 × 10^03^
4	50	5.30 × 10^01^	5.77 × 10^02^	2.97 × 10^03^
5	60	8.16 × 10^01^	6.17 × 10^02^	3.01 × 10^03^
6	60	9.00 × 10^01^	7.47 × 10^02^	3.62 × 10^03^
7	80	2.13 × 10^02^	8.43 × 10^02^	3.88 × 10^03^
8	90	3.09 × 10^02^	2.00 × 10^03^	5.76 × 10^03^

### Temperature analysis

The proposed DM-NSFET based biosensor analyses the impact of temperature variations on drain current at 200 K, 300 K, 400 K and 500 K.

**Fig. 13:**
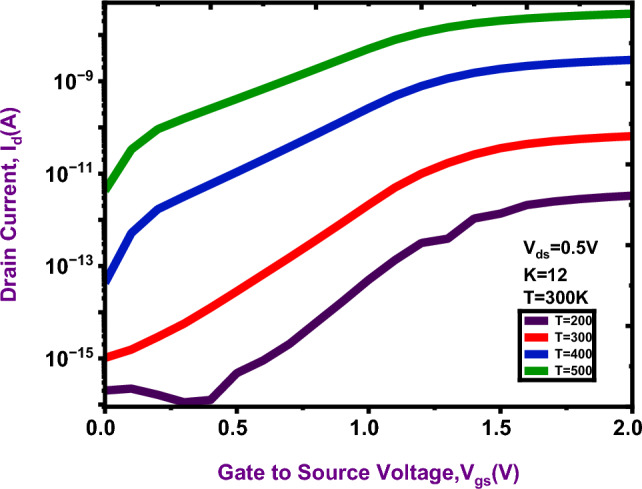
Effect of temperature variation on Proposed device performance.

**Fig. 14:**
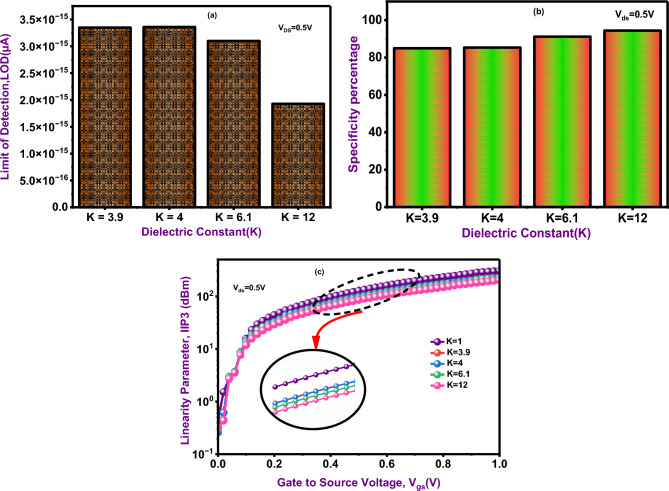
(**a**) Limit of detection (LOD) at different dielectric constant(K) values. (**b**) Specificity Percentage at different dielectric constant(K) values. (**c**) Evaluation of linearity parameter IIP3 considering different dielectric constant(K) values.

Figure 13 shows the dependence drain current on source voltage at different temperatures. An increase in temperature, the ON current increases, this attribute improves the carrier transport and tunnelling, at 500 K the current is high and K = 200 K the current is low. Notably, at 300 K the proposed device exhibits a strong drain current, it demonstrates that this device is reliable and efficient performance under normal condition (T = 300 K). Figure 14 shows the sensing performance of the DM-NSFET for various dielectric constants introduced in the nanocavity. The Limit of Detection (LOD) is reduced as increasing values of dielectric constant is shown in Fig. 14a ^22^. The minimum value is obtained at K = 12, indicating stronger charge interaction and enhanced gate control. The specificity analysis is shown in Fig. 14b demonstrates that the proposed device maintains a high level of specificity with a slight improvement for higher K values due to more pronounced current modulation between target and non-target biomolecules. The specificity percentage is expressed using the following Eq. (10) ^22^. Figure 14c shows the linearity characteristics, where an increase in the dielectric constant leads to improved IIP3 especially around the middle region of the bias, confirming stable and predictable sensing. The Linearity parameter (IIP3) is calculated using the following Eq. (9) ^40^. Finally, the obtained results suggest that higher-K biomolecules significantly strengthen the electrostatic coupling in the proposed DM-NSFET for superior sensitivity, robust specificity, and improved linearity for reliable applications in biosensing^40–43^.

9 $$IIIP3 = \frac{2}{3}*\frac{{g_{m1} }}{{g_{m3} *R_{s} }}$$

10 $$Specificity\; Percentage = \frac{{I_{d} \left( {target} \right) - I_{d} \left( {Non - target} \right)}}{{I_{d} \left( {target} \right)}}$$

**Fig. 15:**
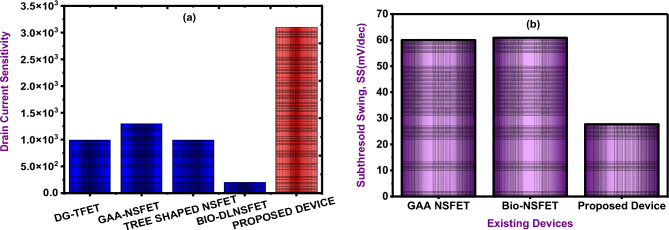
Benchmarking of DM-NSFET biosensor against reported NSFET-based biosensors across key performance metrics (**a**) Drain Current Sensitivity (**b**) Subthreshold Swing(SS).

The comparison of the drain current sensitivity and subthreshold swing(SS) of the proposed DM-NSFET biosensor with previously published NSFET-based biosensors is shown in Table 4 and Fig. 15a. The results confirm the superior sensitivity of proposed device, which is mostly attributed due to its source-drain cavity structure. The proposed device exhibits lower subthreshold swing(SS) compared to existing devices devices like GAA-NSFET and Bio-NSFET, highlighting its superior switching efficiency is shown in Fig. 15b. This cavity increases the probability of tunneling by improving the interaction between biomolecules and the electric field at the source-channel junction. The DM-NSFET biosensor, which was created for the label-free detection of cancer biomolecules, performs noticeably better than previous models. These improved features demonstrate its significant potential to progress biosensing technology.

**Table 4:** Comparison of the DM-NSFET device Performance parameters with existing works.

Devices	Dielectric constant	Sensitivity	Subthreshold Swing
DG-TFET^44^	K = 12	≈10^3^	–
GAA NSFET^45^	K = 10	≈1.3 × 10^3^	60 mV/decade
Tree shaped NSFET^46^	K = 10	≈10^3^	–
Bio-DLNSFET^47^	K = 12	≈2.1 × 10^1^	60.9 mV/decade
Proposed device	K = 12	≈3.1 × 10^3^	mV/decade

## Conclusions

In this work, we have designed and analysed a Vertically Stacked Gate All Around Dielectric Modulated Nano Sheet FET(DM-NSFET) based biosensor for label free detection of cancer biomolecules. Ultra-low voltage operation is made possible by the combination of vertically stacking channels, Cavity All Around the channel at two side of HfO2 dielectric material, which improves the electrostatic control and enable effective band-to-band tunnelling. The proposed device achieves maximum drain current sensitivity of 3.1 × 10^3^, selectivity of 0.46, subthreshold swing (SS) of 27.72 mV/dec for neutral charge biomolecules with dielectric of K = 12. The Maximum drain current sensitivity is obtained at negative charge densities at N_f_ = − 1E12 is 9.14 × 10^3^ at K = 12, positive charge biomolecules with surface charge density N_f_ = − 1E12 is 1.68 × 10^3^ at K = 12. The proposed DM-NSFET can evaluate device performance under partial cavity filling and steric hindrance, demonstrating excellent robustness for real-world sensing environments. The proposed-ON current continuously produces higher sensitivity, ON-current and switching efficiency when compared to existing FET based biosensors. Ultimately, the DM-NSFET biosensor shows itself to be an energy-efficient, CMOS-compatible solution for ultra-sensitive, label-free detection, making it a viable option for next-generation biosensing applications in the environmental, healthcare, and biomedical fields.

## Data Availability

The authors confirm that all data are included within the manuscript.
